# A Wee Lesson in Science Communication

**DOI:** 10.1371/journal.pbio.0020122

**Published:** 2004-04-13

**Authors:** Emma King

## Abstract

The University of Edinburgh encourages its Ph.D. students to participate in a broad programme of science communication activities, designed to enhance public engagement in science

The need for effective communication of research and the promotion of science is more important then ever. Public scepticism of research is high, and the number of students studying science continues to dwindle. In an attempt to combat this, the University of Edinburgh encourages its Ph.D. students to participate in a broad programme of science communication activities, designed to develop transferable skills they can use outside pure research and to enhance public engagement in science (PES).

Included as part of the transferable skills programme, the University hosts a science communication module. The course provides an introduction to the different media, educational, ethical, and political issues surrounding the communication of science to a nonspecialist audience. Initially, the course addresses the presentation of science in written and oral forms. However, as the presentation of science to the public is not simply a practical skill, part of the course is dedicated to the tactical communication of science in society and the relationship between scientists and the media. Finally, students are introduced to ongoing PES opportunities. Any involvement requires the approval of supervisors, to ensure no adverse effects on academic performance. The activities available are diverse and flexible, and they enable students to undertake projects that reflect personal interest and availability.[Fig pbio-0020122-g002] [Fig pbio-0020122-g002]

**Figure pbio-0020122-g002:**
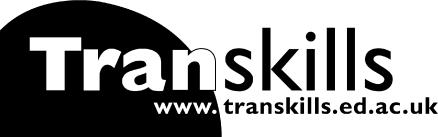


What opportunities are available? For enthusiastic students, membership of the Nikon–University of Edinburgh Post-Graduate Science Communication Team (PGSCT) is possible. The PGSCT requires a commitment of 15 days per academic year to paid science communication projects and events, including compulsory support of SCI-FUN, the University's established science and technology roadshow. The team is recruited annually from students of the Science and Engineering, Medicine, and Veterinary Medicine Colleges and takes a leading role in PES activities. Team members are supported by other graduate students who participate on a more casual basis.

To ensure that a broad spectrum of activities is available to audiences, the PGSCT members are actively encouraged to design and develop their own ideas. An opportunity to do this is provided through the University's Science Zone, at the Royal Museum of Scotland, for the duration of the Edinburgh International Science Festival. From successful workshops, originally piloted at the science festival, several ex-PGSCT members have gone on to establish their own projects. One example of this is the Natural Environment Science Education scheme, which later received recognition through the Royal Society of Edinburgh/Scottish Executive ‘Science in the Community’ Award for 2003. A primary aim of this scheme was to deliver in isolated and remote communities innovative hands-on earth science- and natural science-based activities beyond city venues. For students, developing and presenting workshops are incredibly rewarding and allow them to experience the enthusiasm of participants and fellow presenters.

Some activities are transferred from the Science Zone to the classroom, where students can communicate with children at all stages of their schooling. At primary school age (5–11 years) after-school science clubs, led by graduate students, provide an opportunity for presenters to share with pupils their enthusiasm for science. Alternative activities are directed at secondary school children (12–18 years), with a variety of workshops available across the different scientific disciplines. In particular, The Scottish Institute for Biotechnology Education (SIBE) has been set up within the University to facilitate PES activities such as the popular ‘Green Fingerprinting’ workshop where the principles of DNA fingerprinting are applied to an ecological scenario in the form of a practical activity (Figure 1). The majority of workshops coordinated by SIBE have been funded by the Biotechnology and Biological Sciences Research Council (BBSRC).

**Figure 1:**
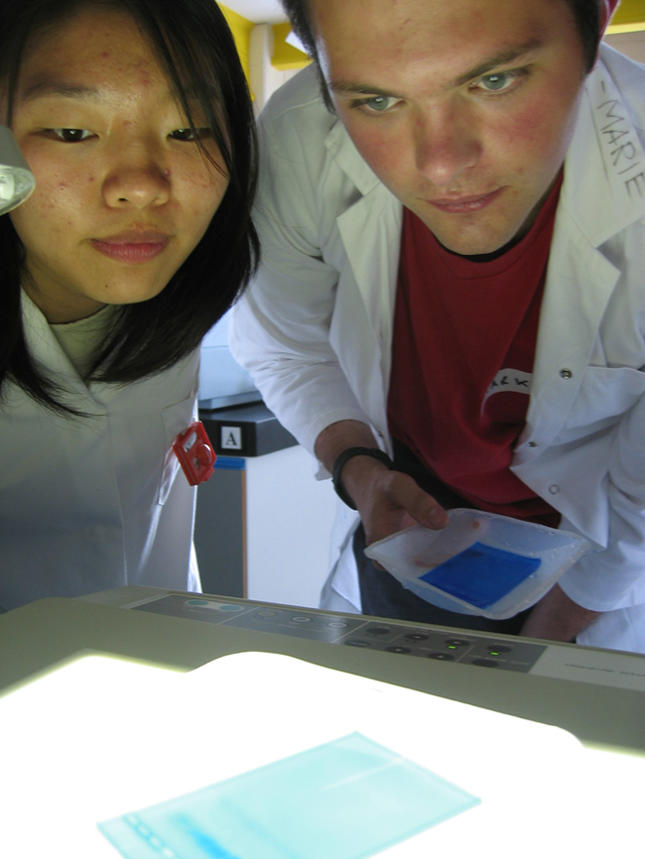
Students Studying a Stained Agarose Gel at a Green Fingerprinting Workshop (Photograph, with permission, by Douglas Robertson)

The presentations may be in person or utilise new technologies such as video conferencing. A BBSRC Dialogue Award has recently been granted to SIBE to design, develop and deliver video-conferences addressing bio-ethical issues surrounding recent advances in biotechnology. Together, the two approaches of in-person and virtual presenting, enhance the schools' curriculum and facilitate dialogue between scientists and the public at a stage where promotion of science can influence the choice of further study and career options.

The promotion of biotechnology within schools does not stop with the encouragement of school children's enthusiasm; SIBE also works closely with organisations such as ‘Science and Plants for Schools’ (SAPS) to assist with continuing professional development of biology teachers through the organisation of training days aimed at curriculum enhancement and the practical application of biotechnology in the classroom. Interested graduate students can co-present the workshops, though they are led by permanent staff. The students introduce their own research to highlight the applications of biotechnology and provide a useful technical resource.

For students who prefer to communicate through the written word, though no guarantee of publication can be given, the University is in a position to highlight science-writing opportunities, usually as a contribution to publications specifically concerned with science communication or within the scientific press. At the Institute of Cell and Molecular Biology at the University a ‘press-gang’ meets on a monthly basis to discuss research carried out in the Institute and generate press releases for publication in the university press and national newspapers where appropriate.

Complementing the University's efforts, many other organisations, such as UK Research Councils and the British Association for the Advancement of Science, endorse graduate students spending time on PES activities. Several schemes have been put in place to facilitate this; Researchers in Residence, the Science and Engineering Ambassadors Scheme and science communication courses. Hosted at the University's science campus—Kings Buildings—‘pgscicom’ provides up-to-date information on opportunities for PES at the University and beyond through regular email communications.

As a PhD student and PGSCT member, I believe the approach of the University of Edinburgh to PES is of benefit to all involved. The combination of training and practical experience provides graduate students with new and valuable skills and opportunities to develop them further. The events and activities enthuse and engage audiences with the presentation of science in an informal but informative manner.

**Figure:**
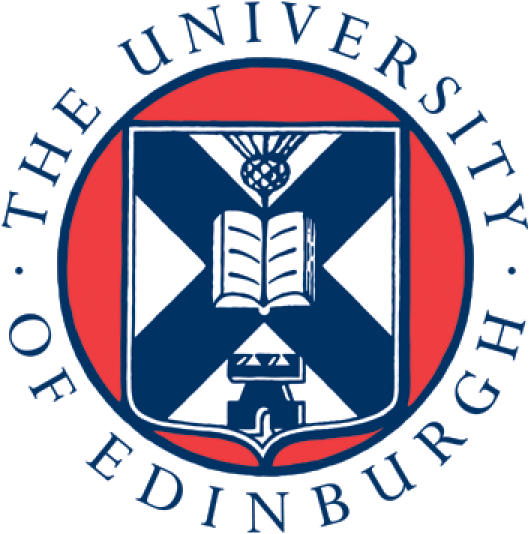


## Abbreviations

BBSRC: Biotechnology and Biological Sciences Research Council
PES: public engagement in science
PGSCT: Post-Graduate Science Communication Team
SIBE: Scottish Institute for Biotechnology Education
